# Current Patterns of Macroalgal Diversity and Biomass in Northern Hemisphere Rocky Shores

**DOI:** 10.1371/journal.pone.0013195

**Published:** 2010-10-07

**Authors:** Brenda Konar, Katrin Iken, Juan José Cruz-Motta, Lisandro Benedetti-Cecchi, Ann Knowlton, Gerhard Pohle, Patricia Miloslavich, Matt Edwards, Thomas Trott, Edward Kimani, Rafael Riosmena-Rodriguez, Melisa Wong, Stuart Jenkins, Angelica Silva, Isabel Sousa Pinto, Yoshihisa Shirayama

**Affiliations:** 1 School of Fisheries and Ocean Sciences, University of Alaska Fairbanks, Fairbanks, Alaska, United States of America; 2 Departamento de Estudios Ambientales, Centro de Biodiversidad Marina, Universidad Simon Bolivar, Caracas, Venezuela; 3 Department of Biology, University of Pisa, CoNISMa, Pisa, Italy; 4 Atlantic Reference Centre, Huntsman Marine Science Centre, St. Andrews, New Brunswick, Canada; 5 Biology Department, College of Sciences, San Diego State University, San Diego, California, United States of America; 6 Department of Biology, Suffolk University, Boston, Massachusetts, United States of America; 7 Kenya Marine and Fisheries Research Institute, Mombasa, Kenya; 8 Programa de Investigación en Botánica Marina, Departamento de Biologia Marina, Universidad Autónoma de Baja California Sur, La Paz, Baja California Sur, México; 9 Bedford Institute of Oceanography, Dartmouth, Nova Scotia, Canada; 10 School of Ocean Sciences, Bangor University, Anglesey, United Kingdom; 11 CIIMAR/CIMAR, Centre for Marine and Environmental Research, University of Porto, Porto, Portugal; 12 Seto Marine Biological Laboratory, Kyoto University, Wakayama, Japan; National Institute of Water & Atmospheric Research (NIWA), New Zealand

## Abstract

Latitudinal gradients in species abundance and diversity have been postulated for nearshore taxa but few analyses have been done over sufficiently broad geographic scales incorporating various nearshore depth strata to empirically test these gradients. Typically, gradients are based on literature reviews and species lists and have focused on alpha diversity across the entire nearshore zone. No studies have used a standardized protocol in the field to examine species density among sites across a large spatial scale while also focusing on particular depth strata. The present research used field collected samples in the northern hemisphere to explore the relationships between macroalgal species density and biomass along intertidal heights and subtidal depths and latitude. Results indicated no overall correlations between either estimates of species density or biomass with latitude, although the highest numbers of both were found at mid-latitudes. However, when strata were examined separately, significant positive correlations were found for both species numbers and biomass at particular strata, namely the intertidal ones. While the data presented in this paper have some limitations, we show that latitudinal macroalgal trends in species density and biomass do exist for some strata in the northern hemisphere with more taxa and biomass at higher latitudes.

## Introduction

Terrestrial systems have generally shown an increase in species numbers from the poles to the tropics [1]. Likewise, early marine studies confirmed this trend [2], [3] and discussed its ecological implications for the marine environment [4]. Since these early studies, others have examined various aspects of latitudinal biodiversity gradients in marine systems, although with varying results, suggesting that while such trends may be general they are not ubiquitous [5]–[14]. Similar to these general latitudinal studies, some studies have focused on macroalgal biodiversity patterns along latitudinal gradients [15]–[22].

Early studies on macroalgae suggested that there is no evidence of a latitudinal trend of increasing species numbers towards the tropics [15], [18]. In fact, areas of both low and high species richness have been identified at sites throughout temperate and tropical waters. Studies since this early work have reported varying results such as increased species richness at mid latitudes and also towards the equator or decreasing species richness towards the equator [5], [16], [22], [23]. A recent literature review covering 387 sites throughout the Atlantic, Indo-Pacific, and Southern Oceans, which spanned 140° of latitude found that in general, temperate oceans tended to have the highest numbers (350–450) of macroalgal genera, particularly between 110° and 160°E longitude [20]. Interestingly, Kerswell [20] also generally found that the number of algal genera had distinct hotspots, namely around Japan and southern Australia. Other studies have identified hotspots in the Mediterranean, the Philippines [18], the Pacific coast of North America [17], the Atlantic European coast [24], and the Caribbean [24]. The current belief is that while lower species richness occurs at the poles, macroalgae generally exhibit variable species richness patterns in different areas [22]. Sometimes these patterns show an increase with latitude, sometimes they decrease, or sometimes they peak at mid-latitudes.

Previous studies on macroalgae have focused on alpha diversity, which examines macroalgal richness within the full extent of a single community, typically homogenizing the various depth and/or intertidal height strata. However, when examining nearshore latitudinal gradients, it is important to consider the intertidal height or water depth from where the samples are taken [25]. This is particularly important for point diversity studies, which focus on a predetermined subset of species from the total site [26]. Since macroalgal species typically occupy particular locations (strata) along a latitudinal gradient, point diversity studies must standardize the strata and the sampling design from which the samples are taken. For example, south-western Iceland, southern Alaska, and the Magellanic region all have recognizable species depth distribution patterns with species diversity increasing seaward in the intertidal [27]. Hence, point diversity samples taken from the high intertidal stratum in one region are not comparable to samples from the low stratum of another region or even of the same region. Similarly, across the Gulf of Alaska, both species richness and abundance/biomass displayed depth strata-related patterns depending on the taxon group being examined [25]. Because of this, it is important to keep intertidal heights and water depths consistent during latitudinal gradient analyses for point diversity.

Most of the previous work on spatial patterns of macroalgal diversity mentioned above was based on non-structured meta-analyses of the existing literature and available species lists. One of the main problems with these previous types of studies is that methodological problems may obscure or artificially impose spatial trends. A potentially more powerful analysis to examine latitudinal gradients would be based on standardized sampling protocols to avoid any biases introduced by varying collection methods. This also would improve diversity-biomass comparisons if data were obtained from the same samples. Using a standardized protocol, however, does introduce its own issues. This is because sites are not similar as far as size of the dominant species or the overall dispersion of the various species. Hence, it would be difficult if not impossible to obtain a true measure of alpha diversity for a number of sites using a standardized protocol. However, point diversity lends itself very well to the use of a standardized protocol because it is only examining a subset of the overall richness within each site.

Another aspect of macroalgal community organization that is sometimes explored is the relationship between different diversity-related attributes. Specifically, the importance of biomass in predicting species richness has been examined [28]–[31]. While the Engelhardt and Ritchie study [30] found higher algal and macrophyte biomass in mesocosms associated with a greater macrophyte species richness, Gough et al. [28] showed that environmental variables explained much more of the variation in potential species richness than biomass. However, when sites exposed to extreme environmental conditions were eliminated from the analysis, biomass became the primary predictor of realized richness. In Portugal, macroalgal species richness was found to be significantly correlated with total biomass on intertidal boulders [31]. An inverse relationship was found in South Africa where high algal biomass and low species richness along the cool and warm temperate region of the coastline was linked to upwelling activity and wave action indices. This influence of upwelling on macroalgal biomass has been described elsewhere [32]. Conversely, low algal biomass and high species richness has been attributed to warmer immersion and emersion temperatures along the sub-tropical region of the coastline [29]. While these latter studies have provided some regional knowledge, there have been few larger scale studies on macroalgal community organization and diversity-related attributes to make any general statements regarding diversity/biomass relationships.

In the current paper, we explore the relationships between macroalgal taxon numbers and their respective biomass with latitude by depth strata using a standardized sampling design. We also determine if correlations exist between the number of macroalgal taxa and biomass with all depths pooled together. We hypothesize that similar to studies on alpha diversity [5], [16], [18], [20], species density [33], as a proxy for point diversity, and macroalgal biomass will show latitudinal trends with higher numbers in mid latitudes. We also hypothesize that using a standardized protocol where species richness data are taken from the same samples as biomass data, we will find that, similar to others [28]–[31], macroalgal species richness will be correlated with total biomass.

## Methods

**Figure 1:**
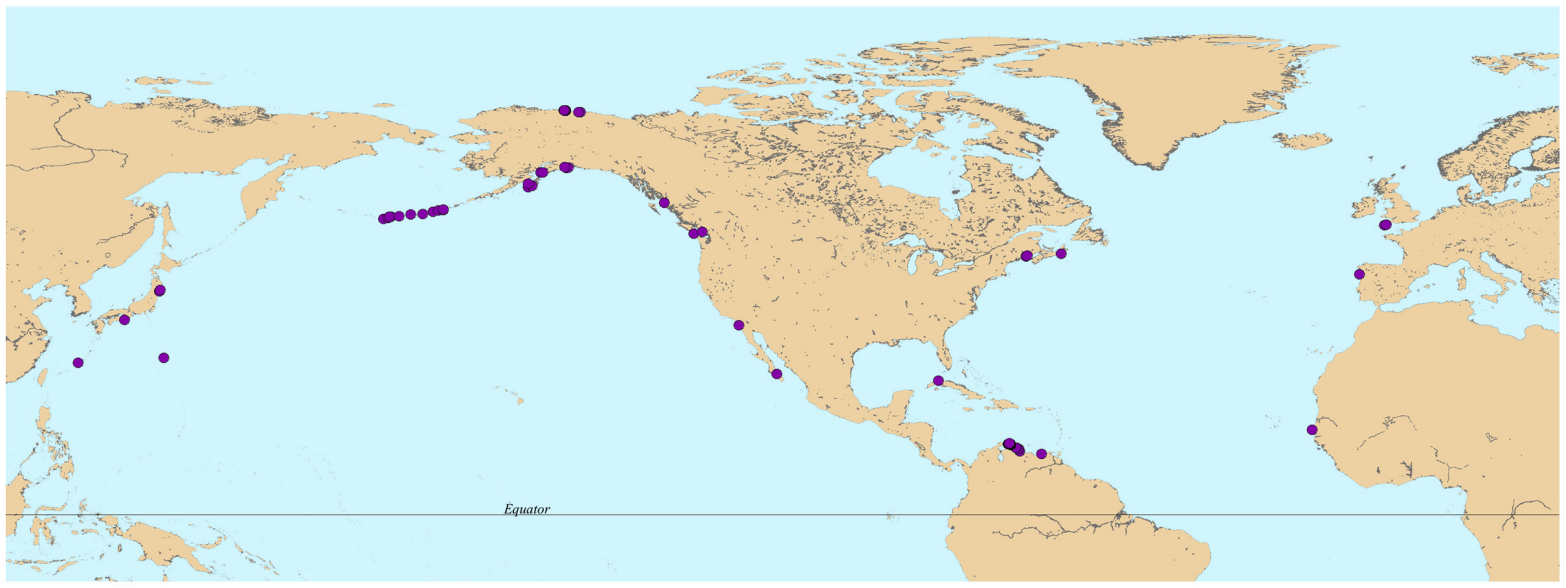
Purple dots refer to the 69 sites sampled. Note that some sites are geographically close together so in some areas dots are overlapping.

Macroalgal communities were sampled at 69 rocky substrate sites from approximately 10°N to 60°N latitude (Figure 1, Supplementary Table S1). Sites were primarily sampled between 2005 and 2009, except in Alaska, USA, where some sites were sampled in 2003. Although a balanced distribution was attempted, not all regions were sampled equally and in many regions sites were spatially clumped. This was an artifact of the location where researchers involved in this program were based. Several important regions, such as Asia were not adequately sampled, while others, such as Alaska were heavily sampled.

Species richness is defined in this paper as point diversity or species density, where richness describes a subset of the community [26]. The use of a standardized protocol is an adequate tool for point diversity comparisons but does not collect absolute site species richness (alpha diversity). For the purposes of this study, we wanted sample numbers and sizes to be equal for our comparisons. All sites were sampled when diversity was thought to be highest for that site (i.e. when annual species were present). Most sites had similar structure with a canopy and understory cover accompanied by algal turf. The standardized protocol used in this study was developed during a workshop for the Natural Geography In Shore Areas (NaGISA) program within the Census of Marine Life initiative [34]. The NaGISA protocol uses a stratified random sampling design at each site in which five replicate random samples are taken along a 30–50 m horizontal transect at the high, mid, and low intertidal strata and 1 m, 5 m, and 10 m below MLLW. Five samples were deemed the best compromise between sufficient replication and practicality of sampling multiple depth strata at each site, especially when the focus of the comparison is point diversity and not alpha diversity. Intertidal heights were determined based on prevailing biobands for that region, such as barnacles, red algae, and brown algae that often typify the high, mid, and low zone, respectively. Not all strata were sampled at all sites because some sites did not have all strata. For example, only the 5 m depth stratum was sampled in the Arctic Beaufort Sea as this is the only depth with hard substrate for macroalgal growth. At each stratum at every site, all macroalgae were removed from within five 50×50 cm quadrats along a horizontal transect line following the stratum. Algae were sorted to the lowest taxonomic level (usually species) and their wet weights determined by taxon using an analytical scale with 1g precision. Taxonomic affinities were verified using the AlgaeBase web site (www.algaebase.org). All encrusting algae were excluded from this study because they could not be completely cleared from the substrate. Data for the five replicate quadrats per stratum were averaged at each site.

Macroalgal assemblages were graphically presented with all strata combined to illustrate general latitudinal trends. Pearson correlations were completed on species numbers and biomass by latitude for the northern hemisphere using StatView (v5.0.1, SAS Institute Inc.).

## Results

**Figure 2:**
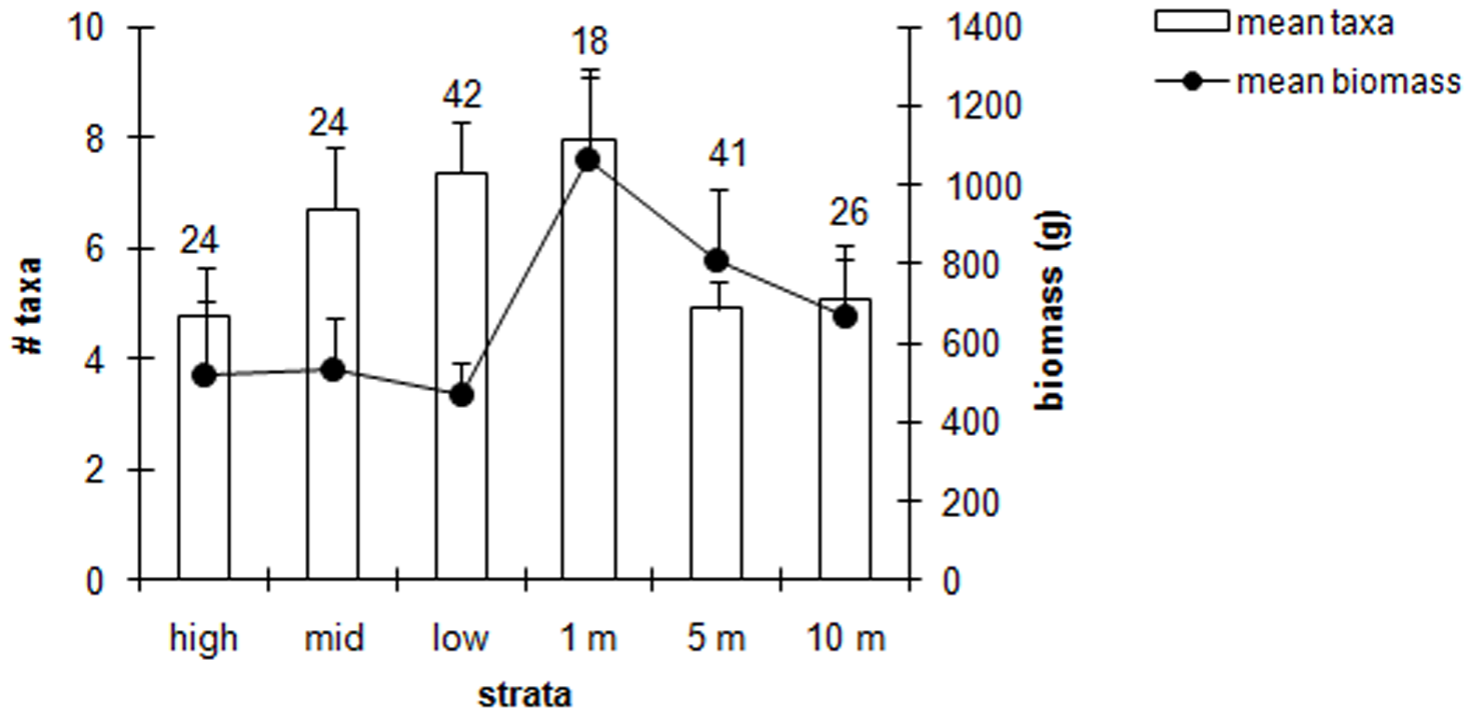
Mean number of taxa and mean biomass (g) per 0.25m^2^ at each stratum. The number above each bar refers to the number of sites sampled for each stratum.

A total of 629 macroalgal species, or higher taxonomic affiliations, were identified during this study. When all sites were combined for each stratum, generally the greatest numbers of taxa were found at the 1 m subtidal depth, with taxon richness decreasing farther into the intertidal and deeper subtidal (Figure 2). In the intertidal, fewer taxa were found in the high than in the low stratum. In the subtidal, there were no noticeable differences between the 5 and 10 m water depths.

Similar to taxon richness, the greatest macroalgal biomass was found at the 1 m intertidal height with biomass decreasing into the intertidal and subtidal strata (Figure 2). However, unlike taxon richness, biomass differences were not observed among intertidal heights or subtidal depths, although a slight trend of decreasing biomass with increasing depth was observed in the subtidal. In general, biomass was generally greater in the subtidal than it was in the intertidal (Figure 2).

**Figure 3:**
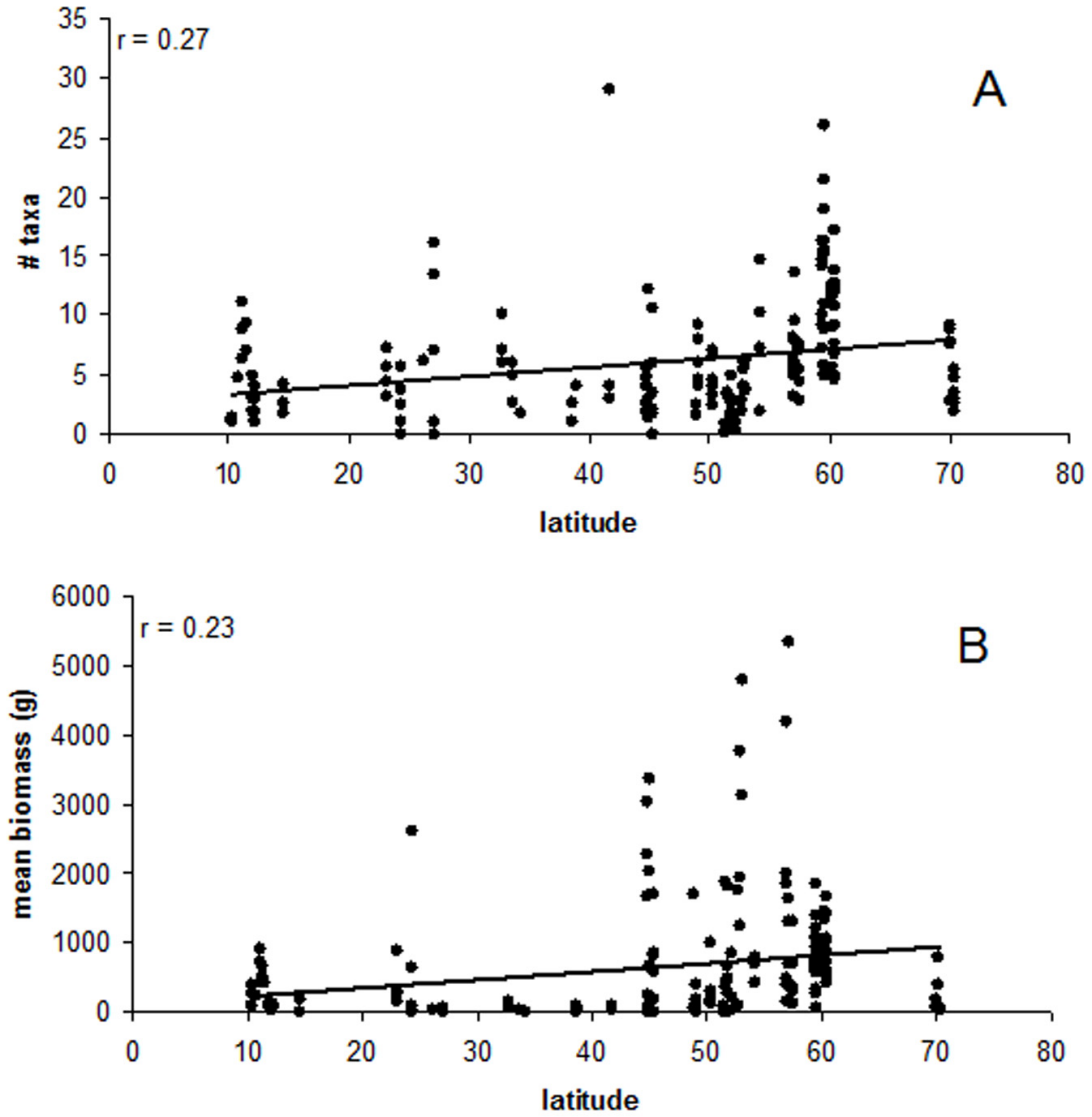
A comparison of A) number of taxa and B) biomass at each latitude (n = 231) showing all strata.

When all strata per site were pooled for a single analysis, significant correlations were not found between latitude and either average taxon numbers or biomass per quadrat (r = 0.27, p = 0.32, n = 176 and r = 0.32, p = 0.19, n = 176 for taxon numbers and biomass, respectively; Figure 3). However, there was a slight trend for both taxa number and biomass to increase at mid latitudes, particularly between 45 to 60 N°.

**Figure 4:**
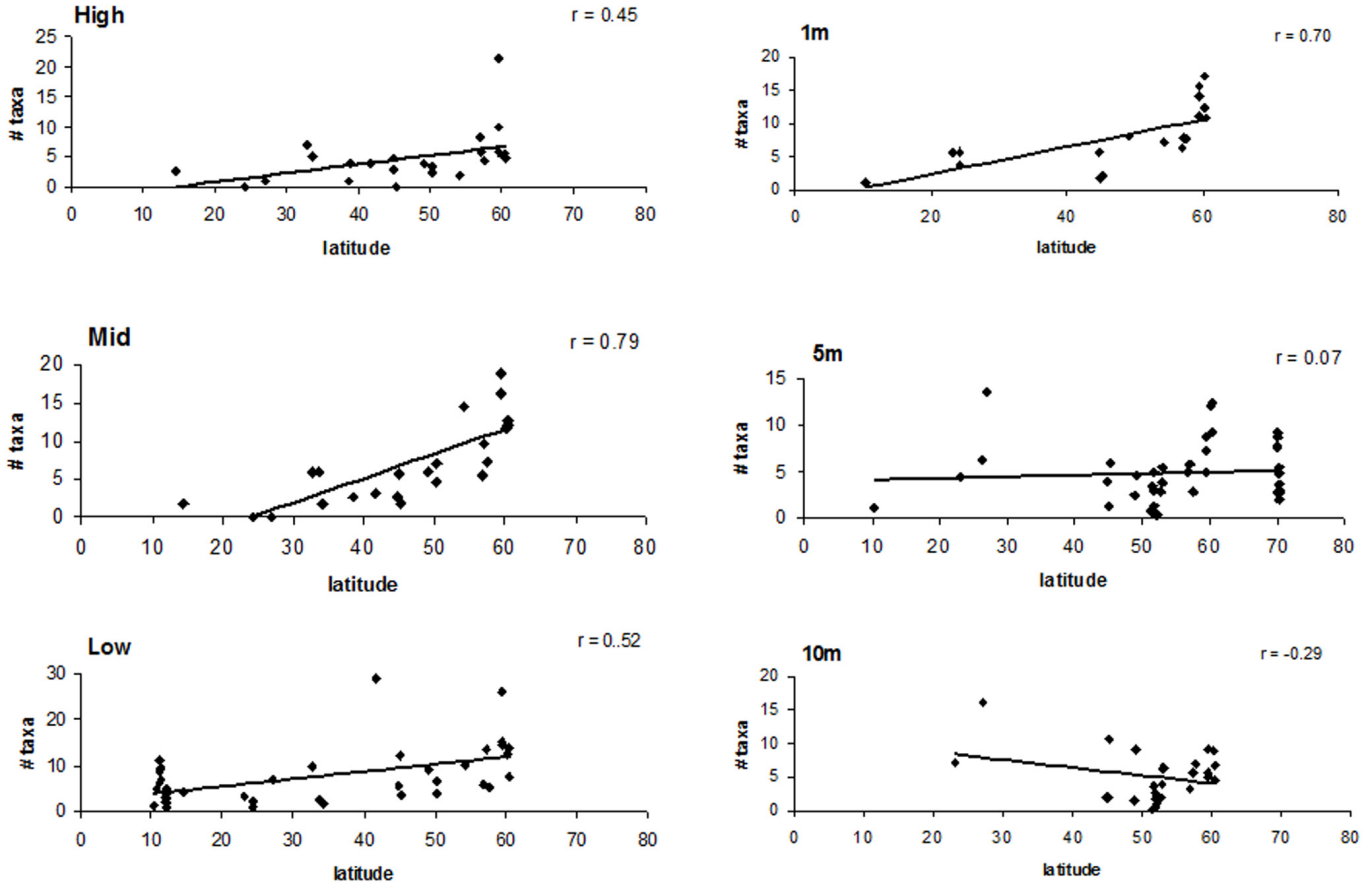
Number of taxa by latitude and for each stratum.

When strata per site were analyzed separately, the highest taxon numbers were typically found at higher latitudes for most strata, specifically around 60°N except at 5 and 10 m where some high values also were seen at around 25°N (Figure 4). Significant positive correlations in latitudinal trends were found for all three intertidal strata and at 1 m, but not for other subtidal strata (Table 1). Some of the highest r values were found in this analysis, with 0.79 and 0.70 in the mid and 1 m strata, respectively.

**Table 1:** Pearson correlations for number of taxa and biomass with latitude.

*# species*						*biomass*					
	r	r^2^	z-value	p-value	n		r	r^2^	z-value	p-value	n
**High**	**0.45**	**0.20**	**2.23**	**0.0259**	**24**	High	0.21	0.04	0.96	0.3362	24
**Mid**	**0.79**	**0.62**	**4.97**	**0.0001**	**25**	**Mid**	**0.46**	**0.21**	**2.33**	**0.0199**	**25**
**Low**	**0.52**	**0.27**	**3.61**	**0.0003**	**42**	**Low**	**0.52**	**0.27**	**3.64**	**0.0003**	**42**
**1m**	**0.70**	**0.49**	**3.35**	**0.0008**	**18**	1m	0.21	0.05	0.84	0.3992	18
5m	0.07	0.01	0.44	0.6577	41	5m	−0.06	0.00	−0.36	0.7172	41
10m	−0.29	0.08	−1.42	0.1549	26	10m	0.24	0.06	1.17	0.2416	26

Bold results refer to statistical significance at α>0.05.

**Figure 5:**
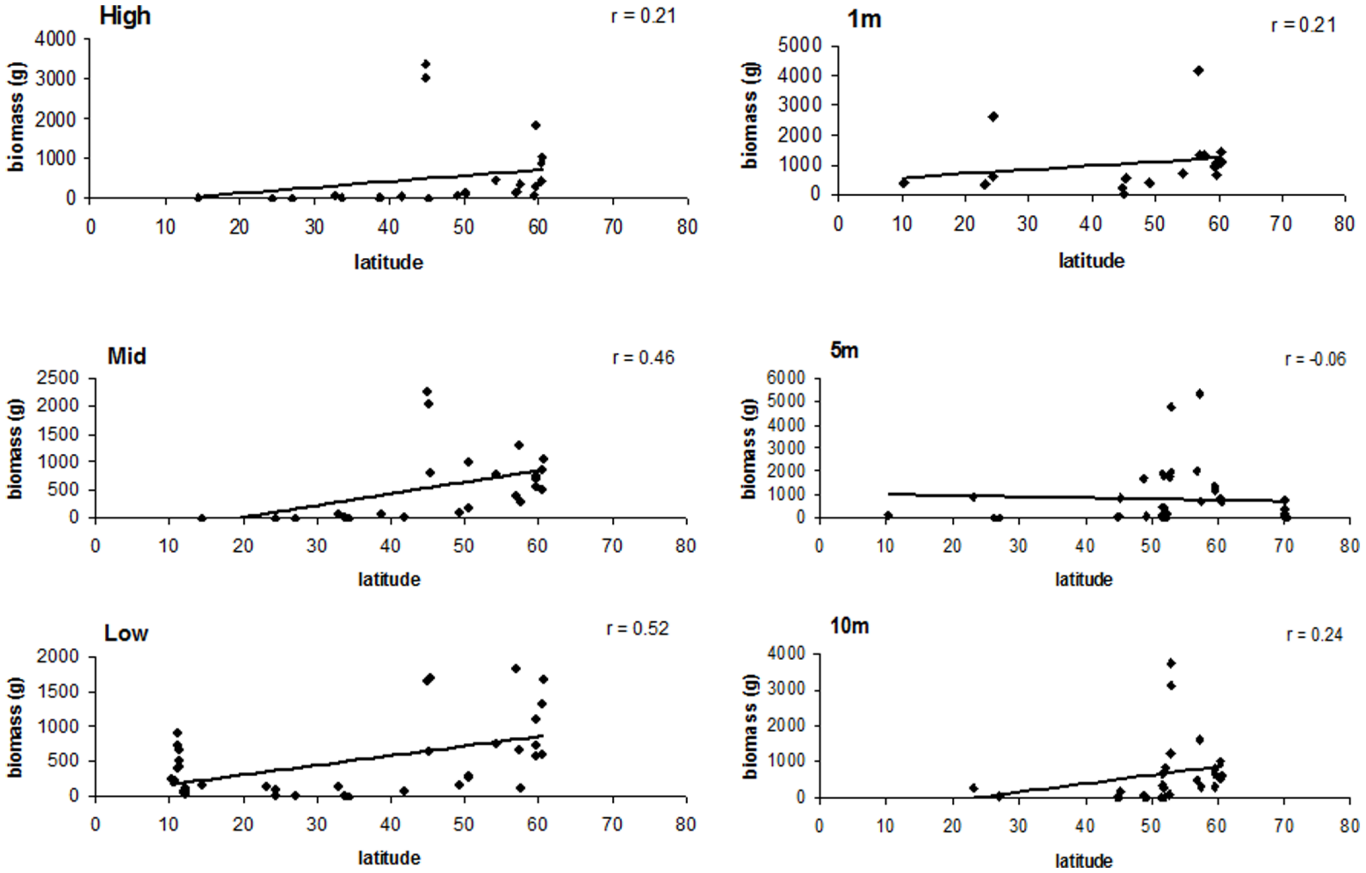
Biomass (g) by latitude and for each stratum.

Overall, highest macroalgal biomass were found at some sites in the high, 1 m, 5 m, and 10 m strata with upwards of 5400 g/0.25 m^2^ at 5 m depth (Figure 5). These high biomass sites were generally around 57°N and occasionally around 45° N (Figure 5). Similar to the number of taxa, biomass in the mid and low strata had significant positive correlations with latitude (Table 1). Overall, r values were relatively low, with 0.46 and 0.52 in the mid and low strata, respectively.

**Figure 6:**
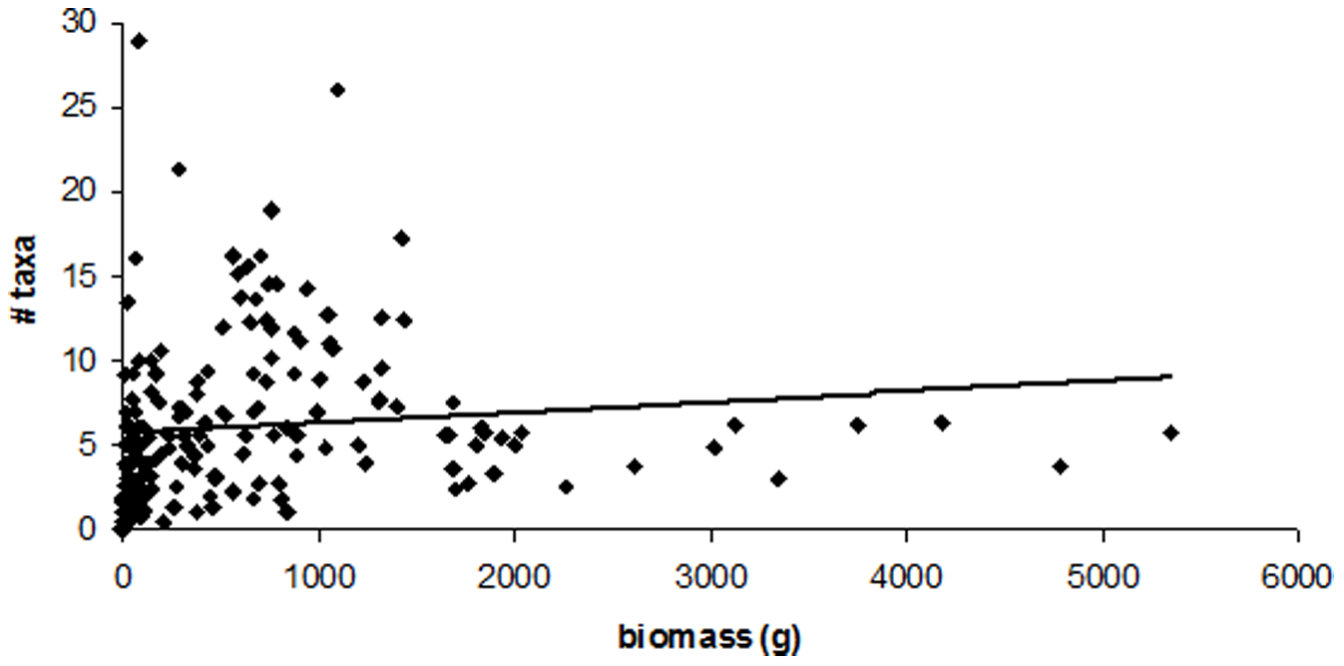
A comparison of macroalgal biomass to number of macroalgal taxa. n = 176, r = 0.12.

Taxon numbers were not correlated with macroalgal biomass (Pearson correlation r = 0.34, p = 0.13, n = 176; Figure 6). Interestingly, the site (in Alaska, USA) with the greatest average biomass (5345 g/0.25 m^2^) was found with an average of 5.8 taxa/0.25 m^2^, while the site (in Portugal) with the most taxa (an average of 29 species/0.25 m^2^) averaged only 76 g/0.25 m^2^ of biomass. Overall the sites with the greatest biomass all had less than ten taxa (Figure 6).

## Discussion

It is difficult to make generalizations about biodiversity in natural systems because of their inherent spatial and temporal variation. However, if generalizations can be proposed, a better understanding of processes and underlying causes may result. This study presents some generalizations regarding macroalgal taxon numbers and biomass along various depth and latitudinal gradients. This paper differs from others in that it examines species density as a proxy for point diversity using a standardized protocol rather than the more typical alpha or beta diversity. It also scrutinized depth strata separately rather than just concurrently examining species richness in the entire nearshore zone at a given site.

One important generalization found in this study was that mean taxon numbers and mean biomass were greatest at the 1 m depth stratum, with lower numbers in the intertidal and deeper subtidal. Similar trends have been seen in eastern Canada, where macroalgal species numbers were negatively correlated with elevation, with fewer species in the higher zones [35]. In the Gulf of Alaska, the macroalgal taxon number also was generally higher at 1 m depth and decreased towards shallower and deeper depths [25]. Although this appears to be a common trend, variation does exist. For example, while macroalgal taxon numbers were greatest in the low intertidal at Kodiak Island (Alaska USA), they were highest at the 5 m stratum in neighboring Prince William Sound just 500 km away [25]. Other local or regional studies examining macroalgal biomass with depth have found similar results to this larger scale study. Macroalgal biomass in the Gulf of Alaska was generally more abundant at the 1 m stratum and decreased with increasing intertidal height and subtidal depth, although study site variation was evident [25]. In Iceland, macroalgal biomass increased seawards from the high intertidal [27], and in California, macroalgal biomass decreased with increasing subtidal depth [36]. Explanations for the high richness and biomass at 1m depth may be related to the special conditions at the interface between the intertidal and the subtidal. On the one hand, since the 1 m stratum is typically only exposed at extreme low tides, it does not experience the harsh conditions that the shallower intertidal strata are subjected to, e.g. desiccation, freezing, and heat, which may lead to lower species richness and biomass in the intertidal. On the other hand, the 1 m stratum experiences higher light conditions than are common at deeper depths and may be less structured by herbivores than the subtidal [37], [38]. This likely optimizes the overall conditions at the 1 m depth stratum for macroalgae, with variations to this pattern based on locally different conditions.

Another general finding was that the number of taxa and average biomass per site decreased in the northern hemisphere from higher to lower latitudes. Peaks were found in the mid latitudes around 45–60°N, with a sharp drop at 70°N in the Arctic (only at the 5 m depth stratum). The only depth stratum that we were able to sample in the high Arctic was 5 m. The drop in taxon numbers at this depth confirms the general observation that macroalgal species richness decreases at the poles [22]. Our observations also support our first hypothesis, that similar to studies on alpha diversity [5], [16], [18], [20], point diversity measured as species density, and macroalgal biomass show latitudinal trends with higher numbers in mid latitudes. This contrasts to a study completed thirty years ago that found large peaks in macroalgal species numbers at 20°N and a smaller peak at 48°N, but no real trend going from north to south [15]. More recently, Kerswell's [20] study on macroalgae found no difference in genus numbers along a global latitudinal gradient but did find hotspots at various latitudes. Other more regional studies have been completed and resulted in various types of trends, including increased species numbers with latitude on the west coast of South Africa and the temperate regions of the Pacific South America, decreased species numbers on the east coast of South Africa and the Atlantic coast of Europe, and mid latitude peaks in the North and Central Americas [5], [16], [19]. Some of the differences among these studies may be attributable to site selection, analyses (alpha versus point diversity), or methodology (literature searches and existing databases in past studies versus a standardized protocol in the present study). Some known northern hemisphere algal diversity hotspots, such as Japan, the Mediterranean, the Philippines, the Atlantic European coast, and the Caribbean [18], [20], [24] were not extensively sampled in the current study. While these latter regions may be actual hotspots attributed to drivers such as sea surface temperature [39], [40], upwellings [31], disturbance [41], [42], spatial heterogeneity [43], or species interactions [44] it could also be that they are simply regions that are better studied.

The use of a standardized protocol allowed us to examine species density separately for each intertidal and subtidal stratum. This analysis showed that there was an increase in taxon number and biomass with latitude in the mid and low zones. There was also an increase in taxon number alone in the high and 1 m strata. The lack of any trends in the subtidal compared to the intertidal strata may be due to the more benign physical conditions in the subtidal than the intertidal. Temperature extremes are greater in the intertidal, as are the problems associated with desiccation. Abiotically generated stressors such as temperature and desiccation typically occur in more unfavorable habitats [45] such as the upper intertidal. It may be that disturbances and other harsh conditions that cause sudden mortality also increase species diversity, although in some circumstances, they may also reduce species diversity [46]–[48]. The more benign conditions found in the subtidal may also play a role in reducing taxon number and biomass variation with depth. It is known that in general, abiotically generated stressors decrease in more favorable environments, such as increasing water cover. However, while abiotic stressors decrease, biotic stresses increase in these more abiotically favorable environments, resulting in competitive exclusion [46], [49], [50]. In this study, more differences may have been found between the mid and low intertidal zones than between 5 and 10 m because of the associated environmental stressors.

In terrestrial communities, species richness is related to community biomass in a “hump-shaped” fashion [51], suggesting two different drivers. At very low biomass, richness is probably limited by abiotic factors causing low survivorship. At very high biomass, it is thought that competitive exclusion may reduce species richness. Some marine studies have found that macroalgal biomass is positively correlated with species richness [30], [31], while others have had conflicting results [28], [52]. The present study found that taxon numbers and biomass were not positively correlated. This does not support our second hypothesis, that similar to other studies [28]–[31], macroalgal species richness is correlated with total biomass. In this study, many sites with very high biomass had very low taxon numbers while other sites with very low biomass had very high taxon numbers and equally, many sites had both very low biomass and very low taxon numbers. The drivers of these relationships are unknown and need to be further investigated.

While the data in this study have limitations, primarily related to small sample sizes and unevenly distributed sites, they have demonstrated that there is a common trend of more taxa and more biomass at mid latitudes in the northern hemisphere, particularly for the intertidal strata. The use of the standardized protocols probably eliminated some of the biases associated with sampling sites using different methodologies making this study powerful in regards to equal effort. The use of species density as a proxy for point diversity was helpful in that it allowed for the comparison of depth strata, rather than looking at the typical all site alpha diversity.

## Supporting Information

Table S1 Macroalgal collection sites. Tidal height indicates the depth strata analyzed from each site. Sites are sorted by ocean.
(0.04 MB XLS)
